# Development and Characterization of a Phthalate-Free Antifungal Soft Denture Lining Material Incorporating a Quaternary Ammonium Monomer

**DOI:** 10.3390/ma19204421

**Published:** 2026-10-21

**Authors:** Patrycja Kula, Izabela Barszczewska-Rybarek, Alicja Kazek-Kęsik, Hubert Knap, Izabella Ślęzak-Prochazka, Grzegorz Chladek

**Affiliations:** 1 Department of Physical Chemistry and Technology of Polymers, Faculty of Chemistry, Silesian University of Technology, Strzody 9 Str., 44-100 Gliwice, Poland; patrycja.kula@polsl.pl; 2 Department of Inorganic Chemistry, Analytical Chemistry and Electrochemistry, Faculty of Chemistry, Silesian University of Technology, Krzywoustego 6 Str., 44-100 Gliwice, Poland; alicja.kazek-kesik@polsl.pl (A.K.-K.); hubert.knap@polsl.pl (H.K.); 3 Biotechnology Centre, Silesian University of Technology, B. Krzywoustego 8 Str., 44-100 Gliwice, Poland; izabella.slezak-prochazka@polsl.pl; 4 Department of Systems Biology and Engineering, Faculty of Automatic Control, Electronics and Computer Science, Silesian University of Technology, Akademicka 16 Str., 44-100 Gliwice, Poland; 5 Materials Research Laboratory, Faculty of Mechanical Engineering, Silesian University of Technology, Konarskiego 18A Str., 44-100 Gliwice, Poland

**Keywords:** antifungal activity, cell viability, mechanical properties, oral candidiasis, physicochemical properties, prosthodontic materials, quaternary ammonium compound, soft denture liner

## Abstract

Soft denture lining materials are widely used to improve patient comfort; however, their susceptibility to fungal colonization and concerns regarding phthalate plasticizer use remain major limitations. This study aimed to develop and evaluate a phthalate-free acrylic soft lining material incorporating 2-phenylethyl benzoate (PhB) as a safer plasticizer and 2-(methacryloyloxy)ethyl-2-decylhydroxyethylmethylammonium bromide (QAHAMA-10), a quaternary ammonium-functionalized monomer, as an antifungal component. The synthesized materials with increasing QAHAMA-10 content (7%, 11%, 15% and 18%) were evaluated for their physico-mechanical properties (Shore A0 hardness, tensile strength, tensile bond strength, water sorption, water solubility, and water contact angle), antifungal activity against *Candida albicans* using adhesion and agar diffusion assays, and cytotoxicity using L-929 fibroblast cells. Incorporation of QAHAMA-10 reduced hardness and surface contact angle while increasing water sorption and solubility; however, tensile strength remained within an acceptable range. The absence of inhibition zones may suggest a predominantly contact-dependent antimicrobial mechanism. All modified materials significantly reduced *C. albicans* adhesion, with no recoverable CFU detected for SLM3 after both 24 h and 28 days of water storage under the applied assay conditions. All formulations exhibited cell viability above 70%, indicating the absence of cytotoxic effects. Among the tested formulations, SLM3 demonstrated the most favorable balance between antifungal activity, cytotoxicity, and mechanical properties.

## 1. Introduction

Soft lining materials (SLMs) are widely used in prosthetic dentistry to improve denture comfort, distribute masticatory loads more evenly, and facilitate the adaptation of dentures to changes in the supporting oral tissues [1,2]. They are particularly beneficial for patients with postoperative tissue defects, chronic mucosal inflammation, severely resorbed alveolar ridges, and elderly individuals with reduced regenerative capacity [3]. To ensure satisfactory clinical performance, SLMs should exhibit adequate and stable properties, including elasticity, bond strength to the acrylic denture base, biocompatibility, and resistance to fungal colonization. SLMs can be classified based on their intended duration of use, clinical purpose, and chemical composition. Based on their intended duration of use, SLMs are categorized into temporary soft lining materials (T-SLMs) and long-term soft lining materials (LT-SLMs). T-SLMs can be further subdivided into tissue conditioners (TCs) and short-term soft lining materials (ST-SLMs). TCs are primarily used for short-term tissue conditioning and cushioning, particularly during tissue healing and postoperative recovery, whereas ST-SLMs are intended to provide cushioning for a longer, but still limited, period of up to 30 days [1,4]. In contrast, LT-SLMs are designed to retain their softness and resilience for several months or years, providing sustained cushioning and stress distribution [1,5]. Based on their chemical composition, SLMs can also be classified as acrylic- or silicone-based materials [4,5]. In the present study, an acrylic-based ST-SLM was developed for short-term use. Soft lining materials may be used in both newly manufactured and existing dentures when cushioning of the denture-bearing tissues or improved denture adaptation is required. Clinical indications include severely resorbed or irregular alveolar ridges, thin or sensitive mucosa, postoperative conditions, tissue healing, and persistent mucosal irritation or pain associated with existing dentures [6]. The frequency of replacement depends on the type of material and its clinical performance. Short-term soft lining materials are intended for use for up to 30 days, whereas long-term materials are intended for use for periods exceeding 30 days [4,5]. Tissue conditioners are intended for very short-term use, typically for several days, and require more frequent replacement due to the relatively rapid loss of their plasticizing components [7]. The replacement of long-term soft lining materials is determined by their clinical condition rather than a predefined period of use and is generally indicated when deterioration in the hygienic condition of the lining or debonding at the interface between the soft lining material and the denture base is observed. Available acrylic-based soft lining materials (A-SLMs) are typically supplied as two-component systems consisting of a powder and a liquid. The “powder” component mainly contains methacrylate polymers, whereas the “liquid” component comprises methacrylate monomers, plasticizers, and, in some formulations, volatile solvents [8]. The composition of the “liquid” is particularly important, as it largely determines the physicochemical and clinical performance of acrylic soft liners.

Plasticizers are essential components of the “liquid” component of SLMs, as they provide softness and elasticity and contribute to the mechanical behavior and clinical performance of relined dentures [7]. Among the plasticizers historically used in acrylic soft liners, phthalate derivatives, such as diisooctyl phthalate (DIOP), have been widely applied due to their favorable physicochemical properties, including low volatility and good compatibility with polymer matrices [9,10]. However, increasing evidence indicates that some phthalates may pose health risks. These compounds are classified as endocrine-disrupting chemicals (EDCs) and have been associated with adverse effects on reproductive system development and hepatic and renal functions [11,12]. Consequently, regulatory agencies, including the European Chemicals Agency (ECHA) and the U.S. Food and Drug Administration (FDA), have raised concerns regarding the use of certain phthalates and emphasized the need for the development of safer alternatives [13]. In response to concerns about phthalate-based plasticizers, attention has shifted toward safer alternatives for medical and dental materials. Among potential candidates, 2-phenylethyl benzoate (PhB), a benzoate ester, has emerged as a promising plasticizer due to its favorable toxicological profile, compatibility with polymer matrices, and potential biodegradability [14]. These characteristics make PhB an attractive alternative to conventional phthalate-based compounds, particularly in applications where long-term contact with biological tissues is expected. Therefore, in the present study, PhB was introduced as a replacement for DIOP in the formulation of a new A-SLM.

Another major challenge in the development of T-SLMs is their susceptibility to fungal colonization. The moist oral environment and the surface characteristics of soft liners promote the adhesion of Candida species and subsequent biofilm formation, making these materials particularly prone to the development of denture stomatitis, one of the most common inflammatory conditions associated with removable dentures [15,16,17]. Therefore, the development of temporary SLMs with intrinsic resistance to fungal colonization has become an important objective in contemporary biomaterials research. To achieve this, a novel polymerizable monomer containing a quaternary ammonium group was synthesized and incorporated into the SLM formulation. Quaternary ammonium compounds exhibit antifungal activity through electrostatic interactions with negatively charged components of fungal cell membranes, resulting in membrane disruption and loss of cellular integrity [18,19]. Unlike conventional antifungal additives that rely on the release of active compounds, such as silver-based agents, essential oils, or antifungal drugs [20,21,22], polymerizable quaternary ammonium monomers become covalently incorporated into the polymer network. Consequently, the antifungal functionality is an integral part of the material structure, minimizing the migration of active compounds into the oral environment while maintaining local antifungal activity [23].

Therefore, the aim of the present study was to develop a phthalate-free acrylic temporary soft lining material (T-SLM) by incorporating 2-phenylethyl benzoate (PhB) as a plasticizer and a polymerizable quaternary ammonium-functionalized monomer providing intrinsic antifungal activity. The study included the synthesis and structural characterization of the monomer as well as the evaluation of the physicochemical properties, antifungal activity, and cytotoxicity of the experimental materials.

It was hypothesized that the combination of PhB with a polymerizable quaternary ammonium-functionalized monomer would result in a temporary soft lining material exhibiting favorable physicochemical properties, effective intrinsic antifungal activity, and acceptable cytotoxicity. Furthermore, increasing the concentration of the antifungal monomer was expected to enhance antifungal efficacy without adversely affecting the physicochemical properties or cytotoxicity of the material.

## 2. Materials and Methods

### 2.1. Chemicals and Reagents

All chemicals used in this study were of analytical grade and used without further purification. The following reagents were obtained from Acros Organics (Geel, Belgium): 1-bromodecane, N-methyldiethanolamine (MDEA), methyl methacrylate (MMA), and ethyl methacrylate (EMA). Ethylene glycol dimethacrylate (EGDMA), N,N-dimethyl-p-toluidine (DMPT), and phenothiazine (PTZ) were purchased from Sigma-Aldrich (St. Louis, MO, USA). Phenethyl benzoate was obtained from Tokyo Chemical Industry (TCI, Tokyo, Japan). Chloroform, potassium carbonate, and toluene were purchased from POCH S.A. (Gliwice, Poland), and ethanol (EtOH) was supplied by Chempur (Piekary Śląskie, Poland). Villacryl Soft powder was provided by Everall7 (Warsaw, Poland).

### 2.2. Monomer Synthesis

The synthesis of 2-(methacryloyloxy)ethyl-2-decylhydroxyethylmethylammonium bromide (QAHAMA-10) was conducted via a three-step procedure, adapted from the method reported by Chrószcz-Porębska et al. [24]. Initially, a transesterification reaction was performed between methyl methacrylate (MMA; 1 mol, 100.12 g) and N-methyldiethanolamine (MDEA; 0.67 mol, 79.85 g), using potassium carbonate (8 wt.%) as the catalyst, phenothiazine (PTZ; 500 ppm) as a polymerization inhibitor, and toluene (407 mL) as the reaction medium. Upon completion of the reaction, the mixture was filtered to remove the solid catalyst and subsequently washed with distilled water (in a 2:1 volume ratio) under magnetic stirring. The aqueous layers were separated using a separatory funnel, and the washing procedure was repeated three times. The collected aqueous phases were then extracted with chloroform (3:1 volume ratio), also in triplicate, to further purify the product. The resulting compound was purified by vacuum distillation (2 mbar), collecting the fraction at 120 °C. This step yielded N,N-(2-hydroxyethyl)methylaminoethyl methacrylate (HAMA) with a yield of 19%.

In the subsequent stage, the isolated HAMA (0.107 mol, 20.0 g) underwent N-alkylation using 1-bromodecane (0.107 mol, 23.67 g), in the presence of PTZ (500 ppm) to prevent unwanted polymerization. The reaction was carried out under mechanical stirring at 82 °C over a period of five days. The N-alkylation step yielded QAHAMA-10 with a reported yield of 100%. No additional purification was performed after the N-alkylation step, as the QAHAMA-10 obtained in this reaction was used directly for the subsequent experiments. Table 1 summarizes the chemicals, reagents, and monomers used in this study, including their functions, sources, and relevant information regarding the synthesis of QAHAMA-10.

The ^1^H and ^13^C NMR spectra of the synthesized QAHAMA-10 were recorded using a 300 MHz NMR spectrometer (UNITY/INOVA, Varian, Palo Alto, CA, USA). For the ^1^H NMR analysis, 256 scans were collected, while ^13^C NMR spectra were obtained using 40,000 scans. Deuterated chloroform (CDCl_3_) served as the solvent, and tetramethylsilane (TMS) was applied as the internal reference.

### 2.3. Formulation and Composition of Experimental Liquid Components for Soft Lining Materials

Five variants of the liquid component were prepared and designated as LC-0 (control), LC-I, LC-II, LC-III, and LC-IV. LC-0 was used as the reference liquid component and was formulated based on the composition of the commercial Villacryl Soft (Everall 7, Warsaw, Poland) material. For clarity, the designation LC-0–LC-IV is used throughout the manuscript to refer to the respective liquid components, whereas SLM0–SLM4 denotes the corresponding final soft denture lining materials obtained by combining each liquid component (LC) with the polymer component (PC). SLM0 contained methyl methacrylate (MMA) and butyl methacrylate (BuMA) as the monomeric components, together with ethylene glycol dimethacrylate (EGDMA) and 2-phenylethyl benzoate (PhB). In contrast, LC-I–LC-IV were modified formulations containing QAHAMA-10, MMA, EGDMA, PhB, and ethanol. BuMA was not present in LC-I–LC-IV. Ethanol was incorporated into the modified formulations to dissolve QAHAMA-10. Across LC-I–LC-IV, the QAHAMA-10 content was progressively increased from 7 to 18 wt%, while the MMA content was correspondingly reduced from 11 to 0 wt%. The EGDMA and PhB contents were maintained at 2 and 74 wt%, respectively, and ethanol was maintained at 6 wt% in all modified formulations. The detailed compositions of all liquid components are presented in Table 2.

### 2.4. Preparation and Polymerization of Experimental Soft Lining Materials

Each of the five liquid components (LCs) was modified by adding 1 wt.% (calculated based on the total mass of the methacrylates used) of N,N-dimethyl-*p*-toluidine (DMPT), which served as a polymerization accelerator (Table 2). Subsequently, five SLMs were prepared by thoroughly mixing the respective LCs with Villacryl Soft powder (PC) at a weight ratio of 1.5 g powder to 1 g liquid. The components were blended for 30 s until a homogeneous paste was obtained. The resulting mixtures were polymerized under pressure at 65 °C for 30 min using a Palamat elite pressure polymerizer (Heraeus Kulzer, Hanau, Germany). The obtained moldings, 2 mm in thickness, were used to fabricate samples for tensile strength, microbiological, and cytotoxicity testing. Additional specimens were prepared in stainless steel molds to ensure consistent shape and dimensions suitable for subsequent analyses.

### 2.5. Biological Tests

#### 2.5.1. Adherence of *Candida albicans* Cells Test

The adhesion of *Candida albicans* (ATCC 2091; BioMaxima, Lublin, Poland) to SLM’s surfaces was evaluated using disc-shaped specimens with a diameter of 10 mm and a thickness of 2 mm. The samples were divided into two groups: the first group was stored in distilled water for 24 h, and the second group for 28 days. After the designated storage period, the specimens were immersed in 1 mL of *C. albicans* suspension in a culture medium (5 × 10^6^ CFU/mL) and incubated at 37 °C for 18 h. The fungal suspension was then removed, and the samples were gently rinsed with 1 mL of sterile water. The specimens were placed in clean centrifuge tubes containing 1 mL of sterile water and subjected to vortexing (1 min, 3000 rpm) to detach the fungi from the surfaces. Subsequently, 100 μL of the obtained suspension was serially diluted in 0.9% NaCl to achieve concentrations of 0.1%, 0.01%, 0.001%, 0.0001%, 0.00001%, and 0.000001%. Then, 100 μL of each dilution was plated onto Sabouraud agar plates (Diag-Med, Warsaw, Poland) and incubated at 37 °C for 18 h. Three repetitions were performed for each specimen. The results were used to evaluate the adhesion of *C. albicans* to the material surfaces. Finally, the number of fungal colonies on the plates was manually counted under transmitted light.

#### 2.5.2. Inhibition Zone of *Candida albicans*

The diameters of the fungal growth inhibition zones (IZDs) were determined for circular samples measuring 10 mm × 2 mm (diameter × thickness). For each formulation, three independent specimens were tested. A fungal suspension (~5 × 10^8^ CFU/mL) was spread over the entire surface of the agar plates, and three SLM samples from each trial were then placed onto the plates. The plates were incubated for 18 h at 37 °C, after which the IZDs were measured.

#### 2.5.3. Cytotoxicity—Alamar Blue Assay

Four specimens were prepared from each material (control and experimental) (*n* = 4) and sterilized under UV light for 45 min in a laminar flow hood (ALPINA Polska, Konin, Poland). Additional FTIR (Perkin-Elmer, Waltham, MA, USA) analysis was performed to assess potential chemical changes following storage and UV sterilization and to evaluate the possible presence of residual ethanol, which could affect the biological test results. The analysis revealed no clear evidence of substantial chemical changes or distinct absorption bands that could be unambiguously attributed to residual ethanol. Disk-shaped specimens (10 mm in diameter and 2 mm in thickness) were individually immersed in 1.76 mL of Dulbecco’s Modified Eagle Medium (DMEM, PAN-Biotech, Aidenbach, Germany) containing 4.5 g/L glucose, L-glutamine, and 3.7 g/L NaHCO_3_. The extraction-medium volume was selected to provide a surface-area-to-volume ratio of 1.25 cm^2^/mL, in accordance with ISO 10993-12 [25]. Extraction was performed for 72 h at 37 °C in a humidified atmosphere containing 5% CO_2_. Following extraction, the media were passed through sterile 0.22 μm nylon syringe filters. Cytocompatibility was assessed using the Alamar Blue reduction assay with L929 mouse fibroblasts (ATCC CCL-1). Cells were maintained in DMEM supplemented with 10% FBS, penicillin/streptomycin, and L-glutamine at 37 °C under humidified conditions with 5% CO_2_. For each measurement, 5000 cells suspended in 50 μL of culture medium were combined with 50 μL of the respective material extract in individual wells of a 96-well plate, yielding a 1:1 (*v*/*v*) extract-to-cell-suspension ratio. For each specimen, measurements were performed at three time points (24, 48, and 72 h), with three technical replicates at each time point. The average of the technical replicates was used as the representative value for the respective specimen and time point. Tissue culture polystyrene (TCPS) served as the negative control; control wells contained 50 μL of cell suspension and 50 μL of fresh culture medium. Wells containing culture medium and Alamar Blue reagent but no cells were used as blanks. After 24, 48, or 72 h of exposure to the material extracts, 10 μL of Alamar Blue reagent was added to each well, and the plates were incubated for an additional 4 h. Absorbance was then recorded at 570 and 600 nm using a Varioskan LUX Multimode Microplate Reader (Thermo Scientific, Waltham, MA, USA). The percentage of Alamar Blue reduction was calculated according to the following equation [26], and the cell viability was expressed as a percentage relative to the TCPS control, calculated by dividing the value obtained for each sample by the corresponding TCPS control value and multiplying by 100%.

### 2.6. Mechanical Tests

#### 2.6.1. Shore A0 Hardness

The hardness of the specimens (*SHA*0) was determined using a Shore A0 durometer (HARDMATIC HH-334, 811-334-10, Mitutoyo, Japan) following ISO 10139-2:2016 [5]. Disc-shaped samples (50 mm in diameter, 6 mm in thickness) were prepared in accordance with the standard. Measurements were performed at 37 ± 1 °C under laboratory conditions after prior conditioning of the specimens in an aqueous environment. Each specimen was placed on a rigid, flat surface, and the durometer indenter was applied perpendicularly to the sample surface. Hardness readings were recorded 5 s after the indenter made contact with the material. Shore A hardness was measured using 15 indentations on three independent specimens. *SHA*0 measurements were carried out at three time intervals: after 24 h, 7 days, and 28 days of storage in water at 37 ± 1 °C.

#### 2.6.2. Tensile Strength

For each material, five Type 4 dumbbell-shaped specimens were prepared in accordance with EN ISO 37 [27]. *TS* was determined using ten independent specimens per formulation. The thickness and width of each specimen were measured before testing. Tensile tests were performed using a universal testing machine (Zwick Z020, Zwick GmbH & Com, Ulm, Germany) at a crosshead speed of 10 mm/min until specimen failure. The ultimate tensile strength (*TS*, MPa) was calculated as the ratio of the breaking force (N) to the original cross-sectional area (mm^2^).

#### 2.6.3. Tensile Bond Strength

Tensile bond strength (*TBS*) between the soft lining materials and the polymethyl methacrylate (PMMA) denture base resin (DBR) was determined following the relevant ISO standard [5]. Rectangular DBR specimens (Vertex Castapres, Vertex-Dental B.V., Zeist, The Netherlands), with dimensions of 15 × 15 mm and a thickness of 4 ± 0.1 mm, were prepared by wet abrasion using P500-grit sandpaper. Before testing, the specimens were stored in distilled water at 37 ± 1 °C for 28 days. The SLM was then introduced into a polyethylene ring (3 mm in height, with an internal diameter of 11 mm) positioned between two DBR plates and polymerized. The assembled specimens were subsequently immersed in distilled water at 37 ± 1 °C for 24 ± 1 h. After mounting the testing handles, the specimens were secured in a universal testing machine (Zwick Z020, Zwick GmbH & Com, Ulm, Germany) and subjected to tensile loading at a crosshead speed of 10 mm/min until failure. *TBS* was determined using five independent specimens per formulation. *TBS* was expressed in MPa and calculated by dividing the maximum force at failure (N) by the initial cross-sectional area of the bonded interface (mm^2^).

### 2.7. Physical Tests

#### 2.7.1. Water Contact Angle

Water contact angle (*WCA*) measurements of the SLM surfaces were conducted using a goniometer (OCA 15EC, Data Physics, Filderstadt, Germany). The assessment was carried out via the sessile drop method on disc-shaped specimens with a diameter of 15 mm and a thickness of 1.5 mm. Measurements were performed on both surfaces of each specimen, with three water droplets applied to each surface. A 4 μL droplet of deionized water was applied to the surface of each sample, and the contact angle was measured immediately after deposition.

#### 2.7.2. Water Sorption and Water Solubility

Water sorption (*WS*) and solubility (*WSL*) were determined in accordance with ISO 10139-2:2016 [5] using five independent specimens per formulation. Disc-shaped specimens (50 mm in diameter, 0.5 mm thick) were first dried to a constant weight (m_1_) and then immersed in distilled water at 37 ± 1 °C for 7 days. After removal from the water, the samples were gently blotted to remove surface moisture and weighed (m_2_). They were subsequently dried again to a constant weight (m_3_). Both drying steps were performed at 37 ± 1 °C in a desiccator (Chemland, Stargard, Poland) containing freshly regenerated silica gel. All measurements were taken using an analytical balance (XP Balance, Mettler Toledo, Greifensee, Switzerland) with a precision of 0.0001 g. *WS* and *WSL* values were then calculated using the following equations:

(1) $$\mathrm{WS}\;\left(\frac{\mu g}{mm^{3}}\right)=\frac{m_{2}-m_{3}}{V}$$

(2) $$\mathrm{WSL}\;\left(\frac{\mu g}{mm^{3}}\right)=\frac{m_{1}-m_{3}}{V}$$

where:

*m*_1_—mass of the samples after initial drying;
*m*_2_—mass of the samples following seven days of water conditioning;
*m*_3_—mass of the samples after re-drying post-immersion;
*V*—original sample volume.

The data were expressed as mean values, and standard deviations (*SD*) were calculated for each set of specimens.

### 2.8. Statistical Analysis

Statistical analysis was carried out using PQStat software, version 1.6.6.204 (PQStat Software, Poznań, Poland). The Shapiro–Wilk test was applied to assess the normality of residual distributions, while Levene’s test was used to verify the homogeneity of variances. Depending on the data, one-way ANOVA with possible F* correction (Brown–Forsythe) followed by Tukey’s HSD post hoc test was used, or alternatively, the nonparametric Kruskal–Wallis test followed by the Dunn–Holm post hoc test or Student’s *t*-test or U Mann–Whitney test for pairwise comparisons (significance level set at α = 0.05).

## 3. Results

### 3.1. Spectroscopic Analysis of Monomer

**Figure 1:**
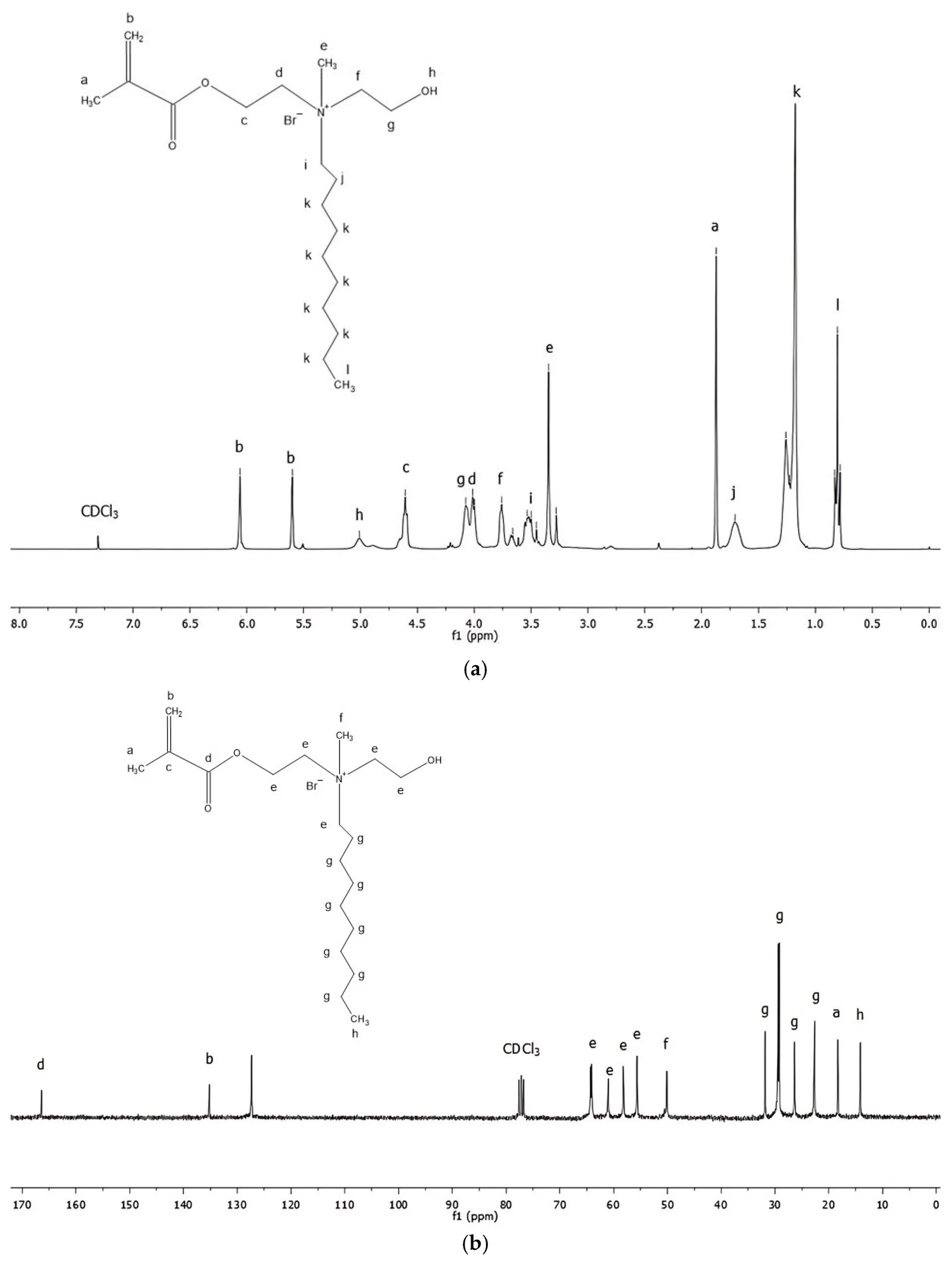
^1^H NMR spectra (**a**) and ^13^C NMR spectra (**b**) of QAHAMA-10. The letter labels indicate the assigned NMR signals, as detailed in Tables S1 and S2.

The QAHAMA-10 monomer was characterized using Proton Nuclear Magnetic Resonance (^1^H NMR) and Carbon Nuclear Magnetic Resonance (^13^C NMR) spectroscopy. Figure 1a presents the ^1^H NMR spectrum of QAHAMA-10. Figure 1b shows the ^13^C NMR spectrum of QAHAMA-10.

The obtained spectra exhibited the characteristic signals corresponding to the chemical structure of QAHAMA-10, confirming the presence of the expected functional groups and carbon environments. The signals observed in the ^1^H NMR and ^13^C NMR spectra were assigned to the corresponding atoms of the QAHAMA-10 structure, and the complete assignments are provided in Tables S1 and S2 in the Supplementary Materials.

### 3.2. Biological Tests

#### 3.2.1. Adherence of *Candida albicans* Cells Test

**Figure 2:**
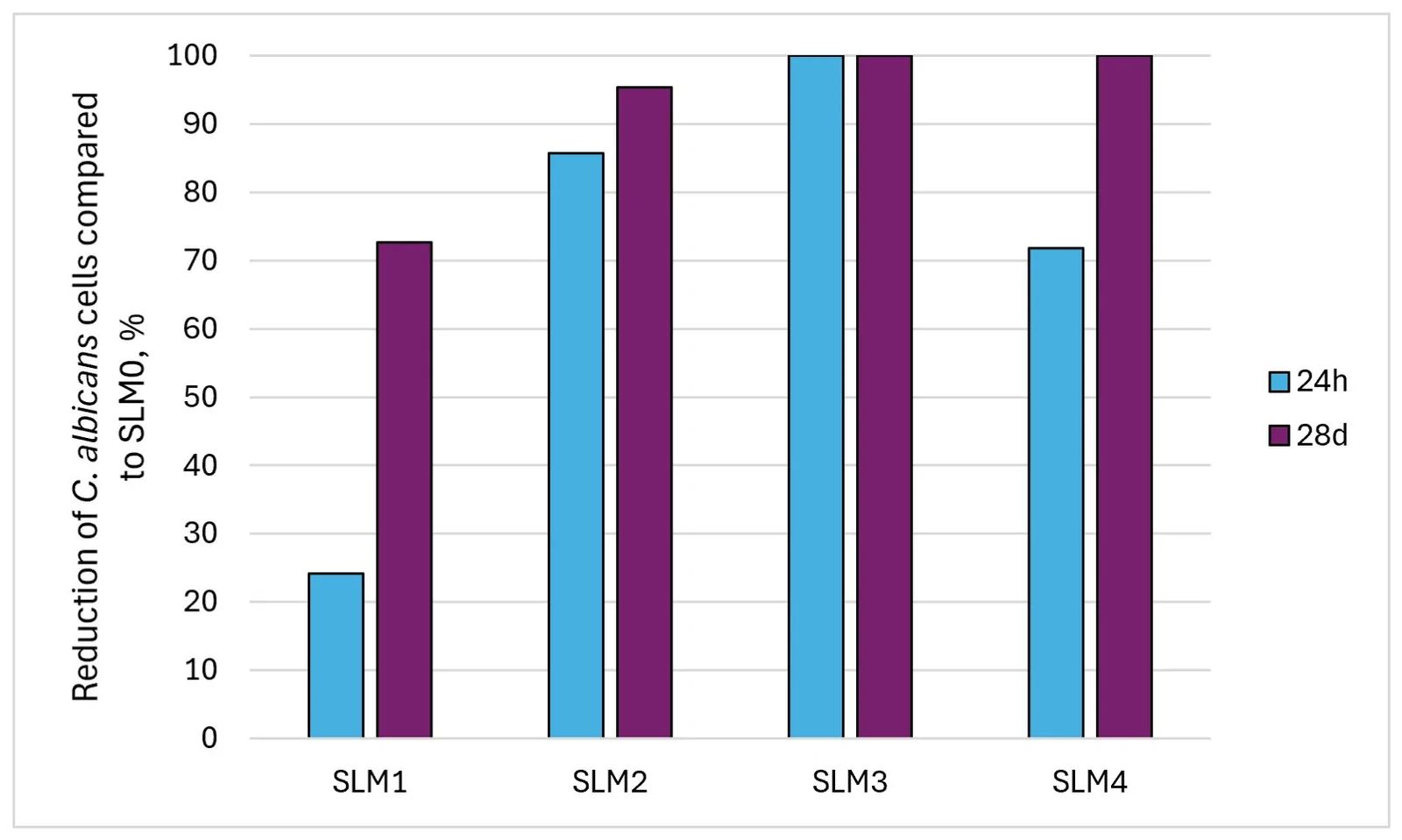
Reduction in *Candida albicans* cells compared to SLM0.

For SLM1–SLM4, the number of viable *C. albicans* cells adhered to the surface (Table 3) decreased relative to SLM0 after both 24 h and 28 days of water immersion (Figure 2).

As shown in Table 3, the median number of viable *C. albicans* cells decreased for all QAHAMA-10-containing formulations compared with SLM0 at both immersion times. After 24 h, the lowest median value was observed for SLM3, for which no viable cells were detected. After 28 days, no viable cells were detected for SLM3 and SLM4, while SLM2 also showed a substantial reduction in the number of viable cells compared with SLM0. After 24 h of water storage, the QAHAMA-10-modified materials exhibited lower median CFU values than the reference material (SLM0); however, the Kruskal–Wallis test did not reveal statistically significant differences among the groups (*p* = 0.0785). After 28 days, statistically significant differences among the formulations were observed (Kruskal–Wallis test, *p* = 0.0131). At this time point, the median CFU values were zero for SLM2, SLM3, and SLM4. Notably, no detectable CFUs were recorded for SLM3 and SLM4 after 28 days of water storage.

The reductions were 24% and 73% for SLM1, 86% and 95% for SLM2, 100% at both time points for SLM3, and 72% and 100% for SLM4, respectively. The theoretical detection limit of the assay was 100 CFU/mL of the recovered suspension, corresponding to 100 CFU per specimen, based on plating 100 μL of the highest-concentration dilution tested (10^−1^) and a recovery volume of 1 mL.

#### 3.2.2. Inhibition Zones of *Candida albicans*

**Figure 3:**
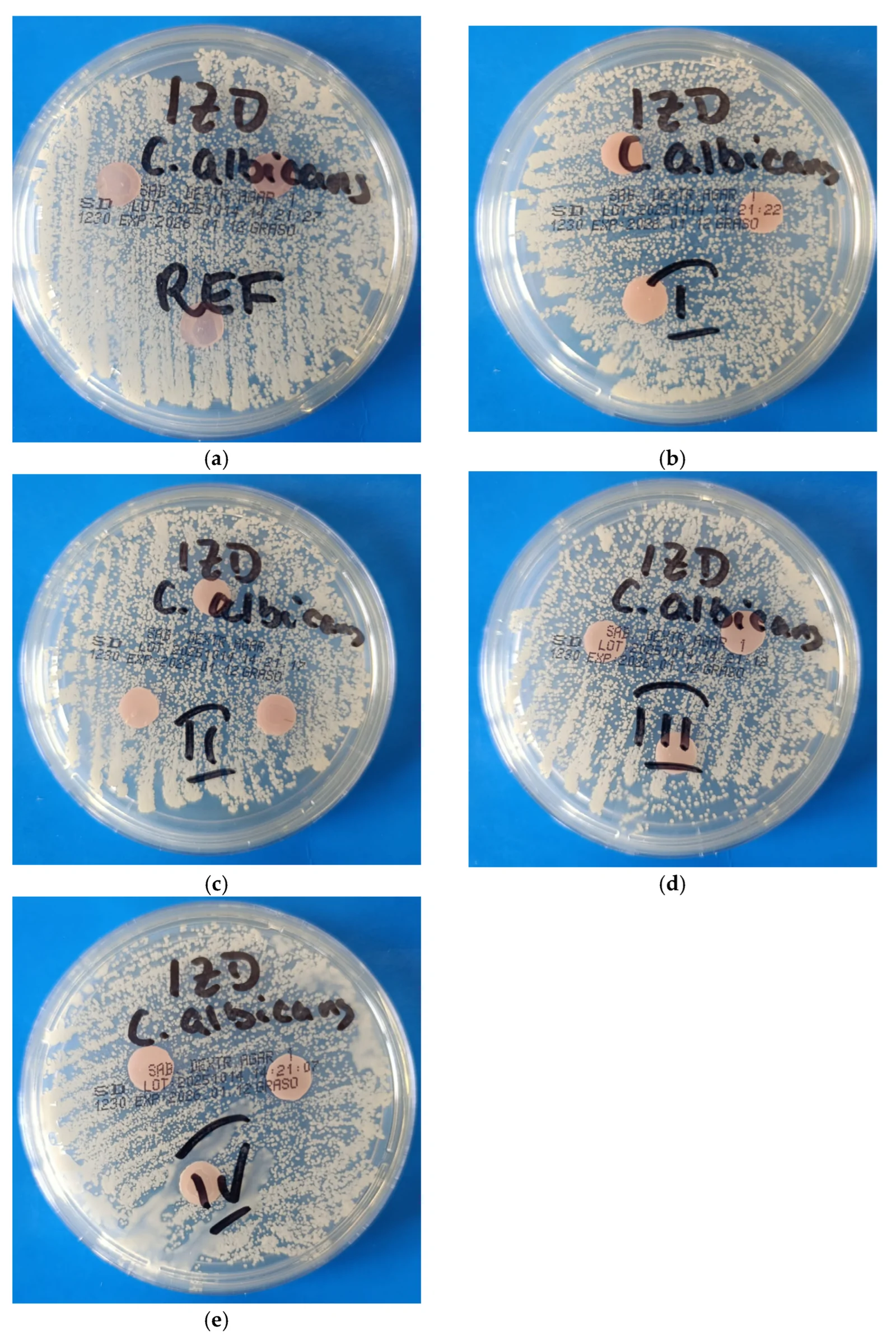
The images of the plates showing the absence of inhibition zones: (**a**) SLM0; (**b**) SLM1; (**c**) SLM2; (**d**) SLM3; and (**e**) SLM4.

All tested SLM samples (SLM0–SLM4) showed no observable inhibition of *C. albicans* growth under the experimental conditions, as shown in Figure 3.

#### 3.2.3. Cytotoxicity—Alamar Blue Assay

**Figure 4:**
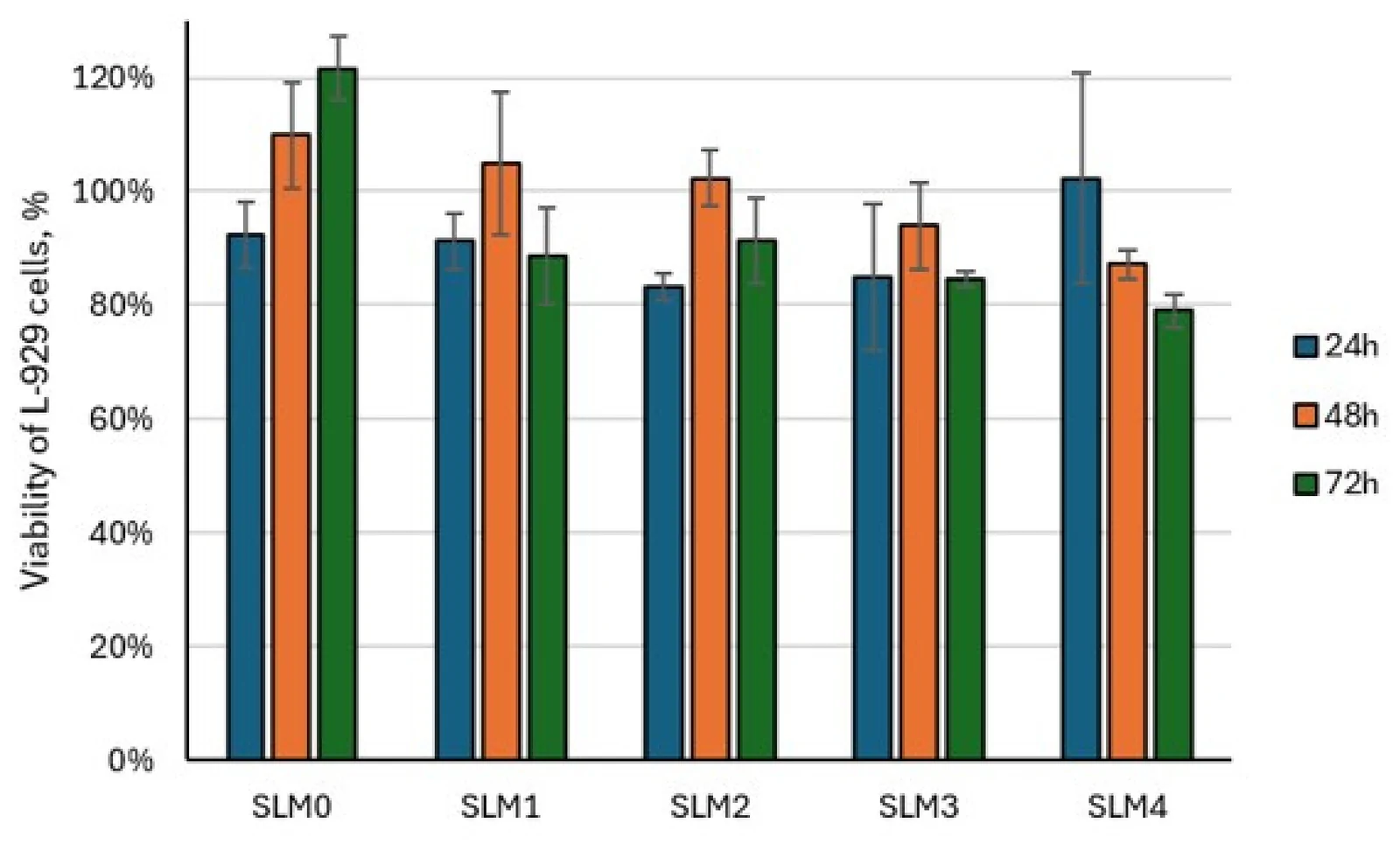
Cytotoxicity evaluation of SLMs using the AlamarBlue assay after 24, 48, and 72 h of incubation.

The results of the L-929 cell viability assessment are presented in Figure 4. Cell viability remained above 70% in all analyzed groups (SLM0–SLM4), regardless of the incubation time. The type of material significantly affected cell viability after 24 h (*p* = 0.0321), 48 h (*p* = 0.0103), and 72 h (*p* = 0.0147) (Table 4). However, the post hoc test did not reveal significant pairwise differences after 24 h, whereas after 48 h, a significant difference was found only between SLM0 and SLM4.

For the SLM0 sample, cell viability gradually increased from 92% after 24 h to 110% after 48 h and 122% after 72 h of incubation, with a significant effect of incubation time (*p* = 0.0009). SLM1 showed viability values of 91%, 105%, and 89% after 24, 48, and 72 h, respectively; the effect of incubation time was not significant but approached the significance threshold (*p* = 0.0706; α = 0.05). For SLM2, viability values of 83%, 102%, and 91% were recorded after 24, 48, and 72 h, respectively, with a significant effect of incubation time (*p* = 0.0023). SLM3 exhibited viability values of 85%, 94%, and 85%, respectively, with no significant effect of incubation time (*p* = 0.2756). In contrast, SLM4 showed viability values of 102%, 87%, and 79% after 24, 48, and 72 h, respectively, and the effect of incubation time was significant (*p* = 0.0392).

### 3.3. Mechanical Tests

#### 3.3.1. Shore A0 Hardness

**Figure 5:**
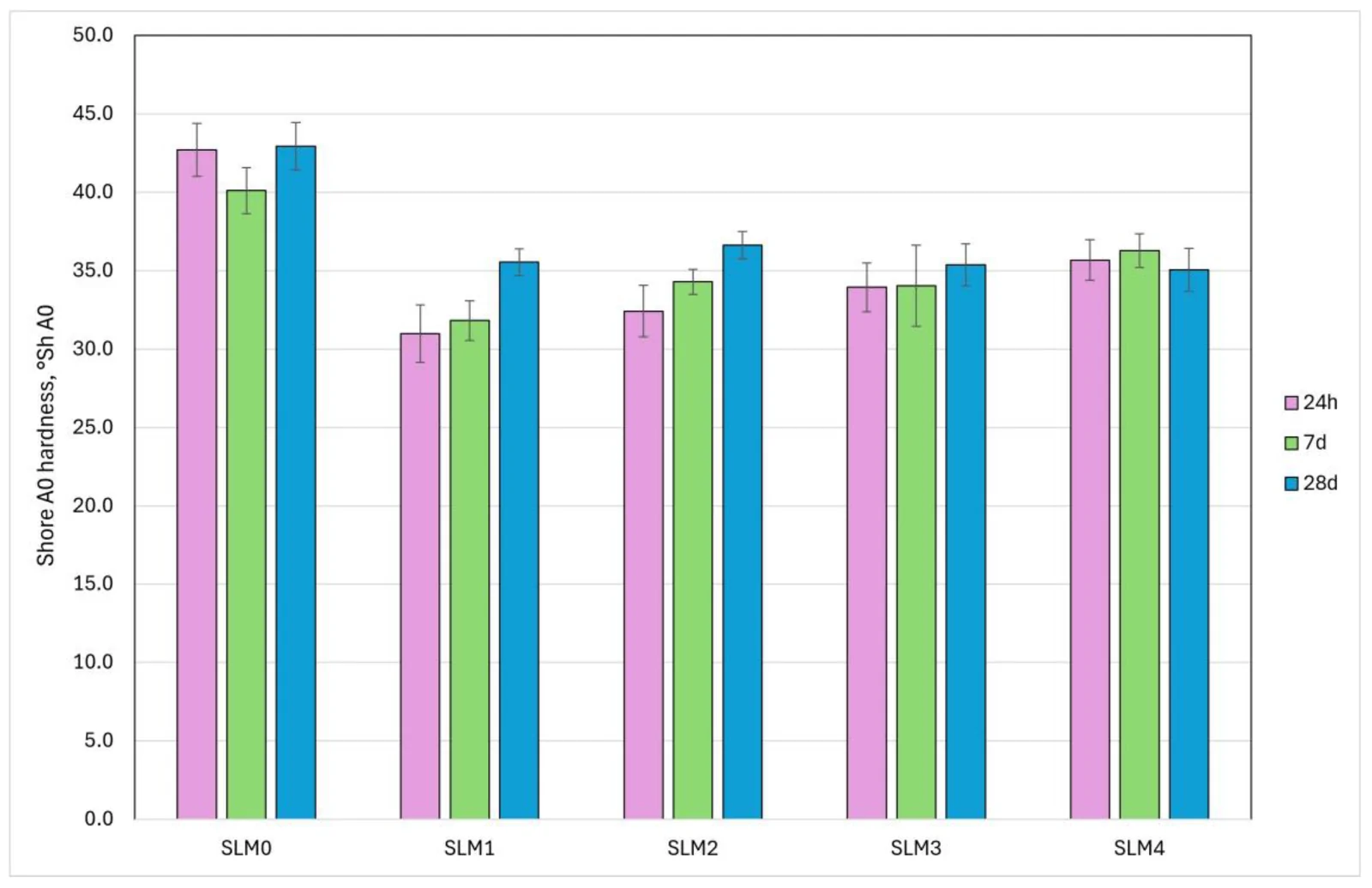
Mean hardness values with standard deviations for SLMs.

Mean Shore A0 hardness (*SHA*0) values are presented in Figure 5, while the statistical comparisons are summarized in Table 5. After 24 h of storage, the mean *SHA*0 values ranged from 31.0 °Sh A0 for SLM1 to 42.7 °Sh A0 for SLM0. After 28 days, the values ranged from 35.1 °Sh A0 for SLM4 to 42.9 °Sh A0 for SLM0.

Throughout the entire experimental period, SLM0 exhibited significantly higher Shore A0 hardness than all experimental materials (SLM1–SLM4; *p* < 0.0001). This relationship remained consistent at all evaluation time points, indicating that the reference material maintained the highest hardness regardless of the storage period. Consequently, all experimental formulations were characterized by lower hardness than SLM0.

Regarding the changes over time, the materials exhibited different patterns of hardness stability. SLM3 was the only material that did not show statistically significant changes in Shore A0 hardness during the entire storage period (*p* = 0.1517), demonstrating the highest hardness stability. In contrast, SLM4 exhibited a significant decrease in hardness compared with its baseline value. Conversely, SLM1 and SLM2 showed a significant increase in Shore A0 hardness during storage, indicating that storage time affected the mechanical behavior of these experimental materials. Overall, most materials underwent significant changes in Shore A0 hardness during the observation period, although the magnitude and direction of these changes depended on the material composition.

#### 3.3.2. Tensile Strength of SLMs

**Figure 6:**
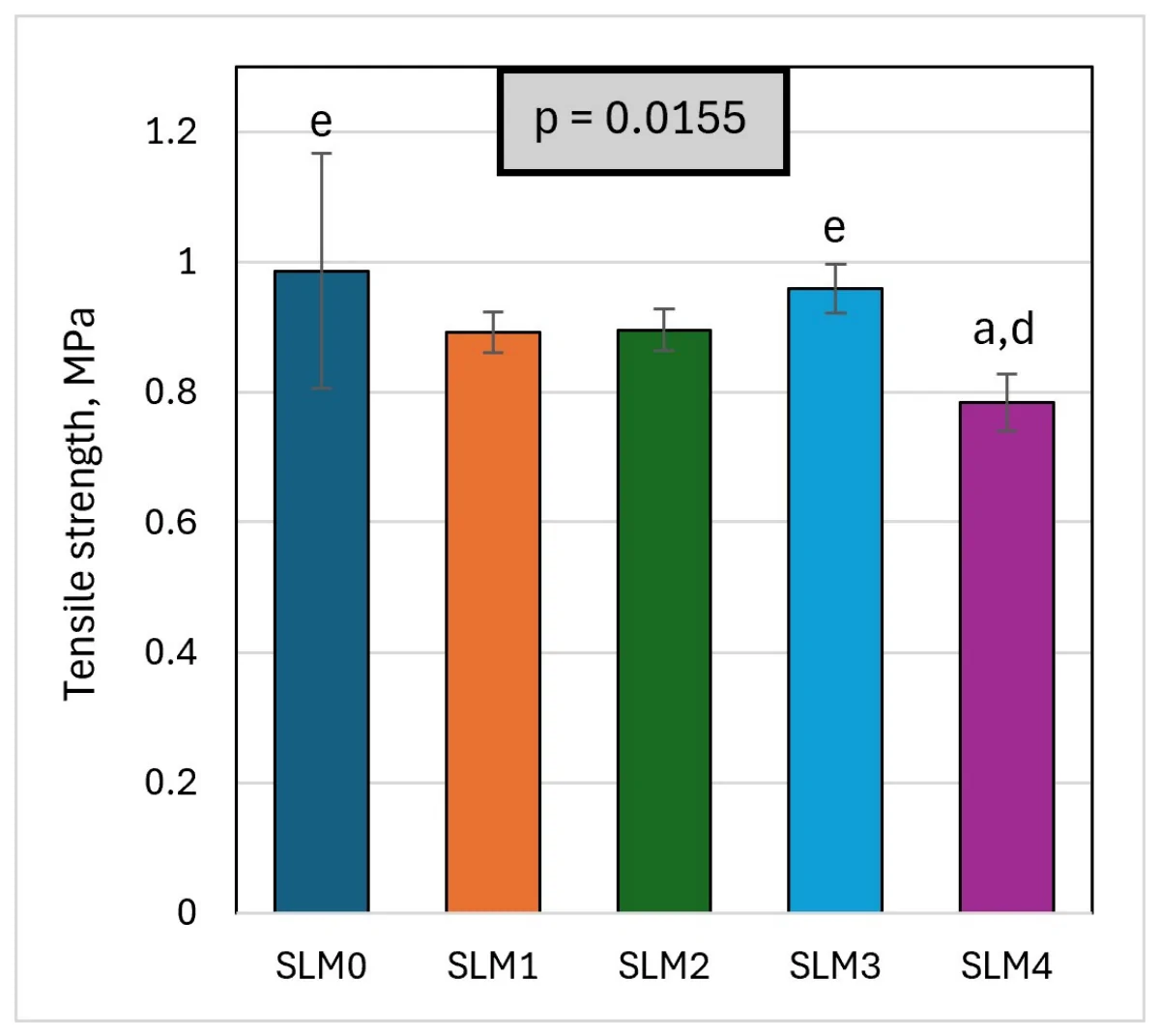
Mean tensile strength values with standard deviations for the SLMs. Lowercase letters above the bars indicate statistically significant differences (Tukey’s HSD, α = 0.05). Each letter corresponds to a material group (a = SLM0, d = SLM3, e = SLM4), indicating a significant difference between the marked bar and the corresponding material.

The introduction of QAHAMA-10 led to a reduction in the mean *TS* values (Figure 6). The maximum *TS* was observed for the reference material SLM0 (0.99 MPa), while the minimum value was recorded for SLM4 (0.79 MPa). The statistical analysis confirmed significant differences between the tested formulations (*p* = 0.01546). However, relative to the unmodified material (SLM0), a statistically significant decrease in *TS* was detected only for SLM4. In addition, SLM3 showed a significantly higher *TS* value compared with SLM4, demonstrating that the effect of QAHAMA-10 incorporation on tensile strength depended on the formulation.

#### 3.3.3. Tensile Bond Strength of SLMs

**Figure 7:**
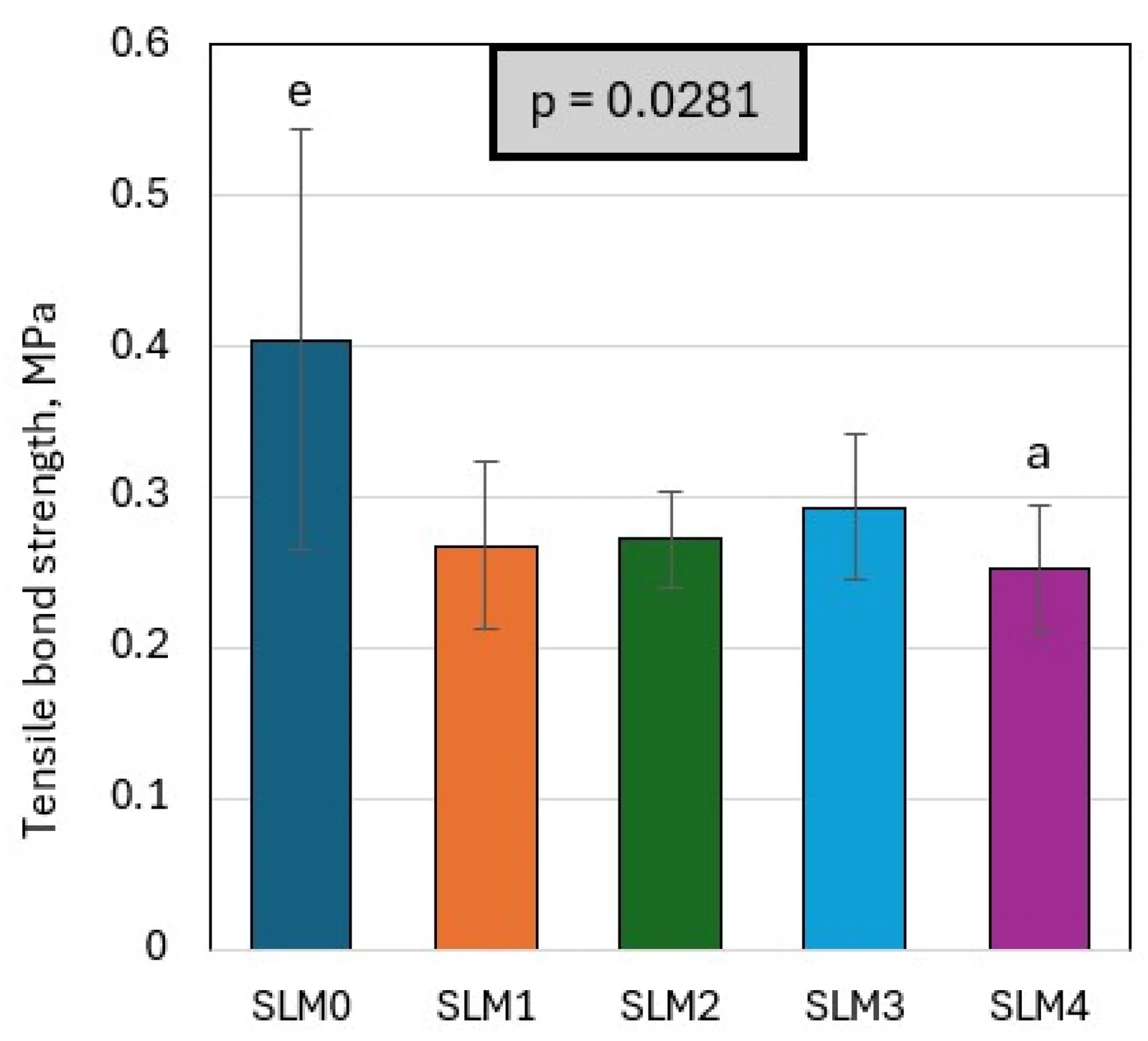
Mean tensile bond strength values with standard deviations for the SLMs. Lowercase letters above the bars indicate statistically significant differences (Tukey’s HSD, α = 0.05). Each letter corresponds to a material group (a = SLM0, e = SLM4), indicating a significant difference between the marked bar and the corresponding material.

The mean *TBS* values decreased following the incorporation of QAHAMA-10 (Figure 7). The highest mean *TBS* was observed for the reference material SLM0 (0.40 MPa), whereas the lowest value was recorded for SLM4 (0.25 MPa). One-way ANOVA revealed statistically significant differences among the tested formulations (*p* = 0.0281). However, post hoc Tukey’s test showed that a statistically significant decrease relative to SLM0 occurred only for SLM4. No statistically significant differences were observed between the remaining pairs of formulations.

### 3.4. Physical Tests

#### 3.4.1. Water Sorption and Water Solubility

**Figure 8:**
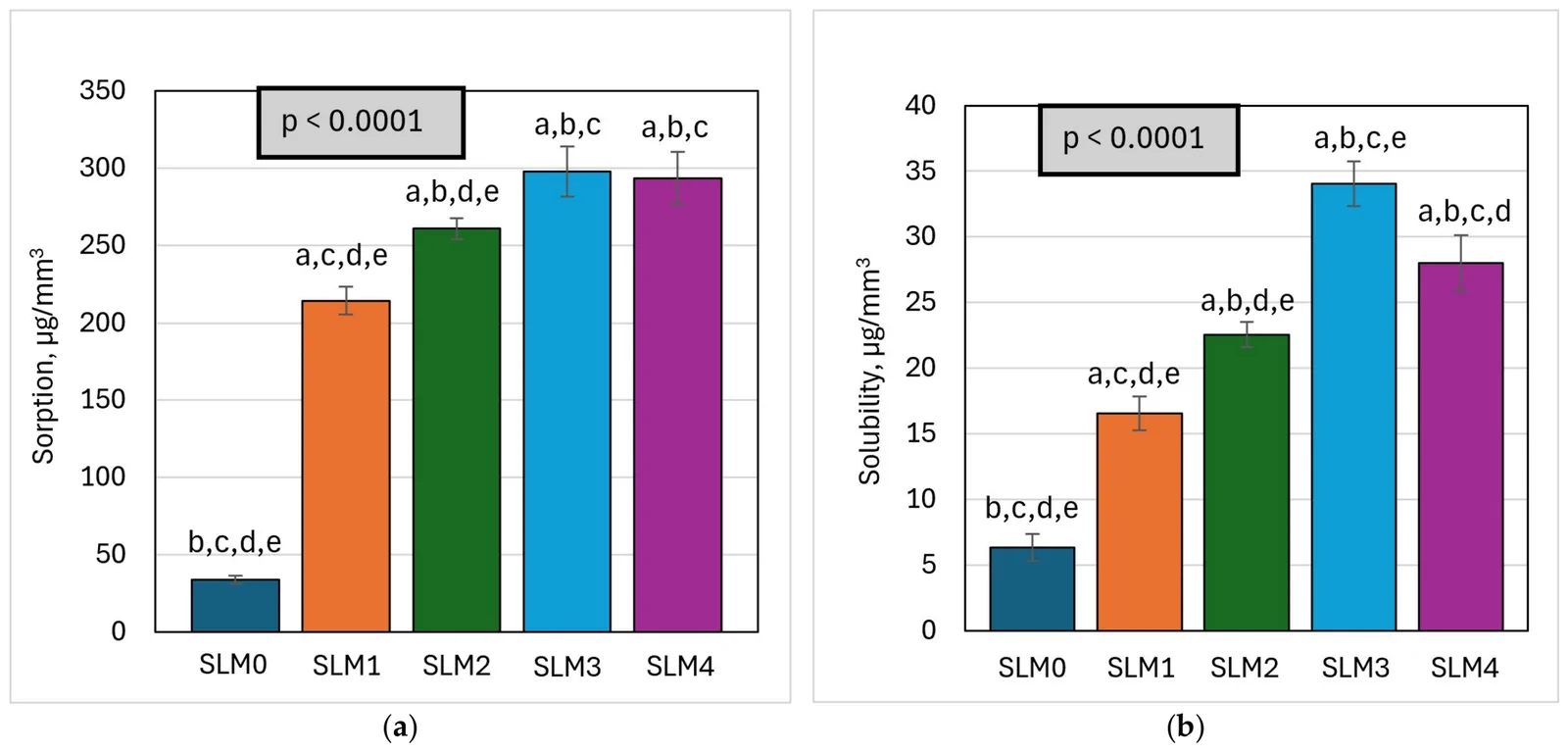
Mean sorption (**a**) and solubility (**b**) values with standard deviations for the tested SLMs. Lowercase letters indicate statistically significant pairwise differences (Tukey’s HSD, α = 0.05). Each letter corresponds to a material group (a = SLM0, b = SLM1, c = SLM2, d = SLM3, e = SLM4) and indicates a significant difference between the marked bar and the corresponding material.

The mean values of *WS* and *WSL* are presented in Figure 8. The incorporation of the experimental monomer had a statistically significant effect on both *WS* and *WSL* values of the SLMs (*p* < 0.0001). The mean values of both parameters were higher for all experimental materials compared with the control material, which exhibited *WS* and *WSL* values of 33.8 μg/mm^3^ and 6.3 μg/mm^3^, respectively. The mean *WS* and *WSL* values were 214.4 μg/mm^3^ and 16.6 μg/mm^3^ for SLM1, 261.0 μg/mm^3^ and 22.6 μg/mm^3^ for SLM2, 297.9 μg/mm^3^ and 34.1 μg/mm^3^ for SLM3, and 293.5 μg/mm^3^ and 28.0 μg/mm^3^ for SLM4, respectively.

#### 3.4.2. Water Contact Angle

**Figure 9:**
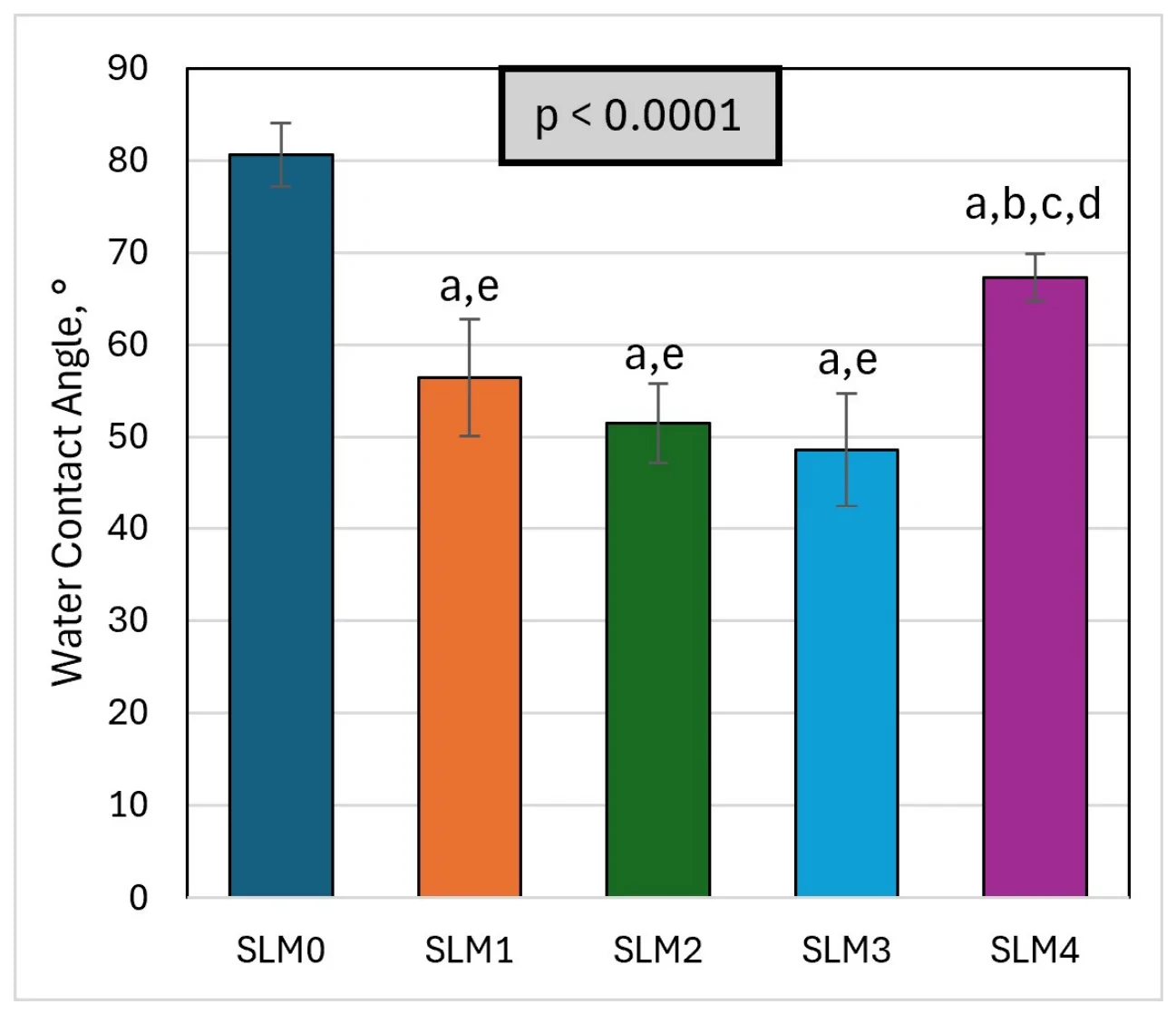
Mean water contact angle values with standard deviations for the SLMs; Lowercase letters indicate statistically significant pairwise differences (Tukey’s HSD, α = 0.05). Each letter corresponds to a material group (a = SLM0, b = SLM1, c = SLM2, d = SLM3, e = SLM4) and indicates a significant difference between the marked bar and the corresponding material.

**Figure 10:**
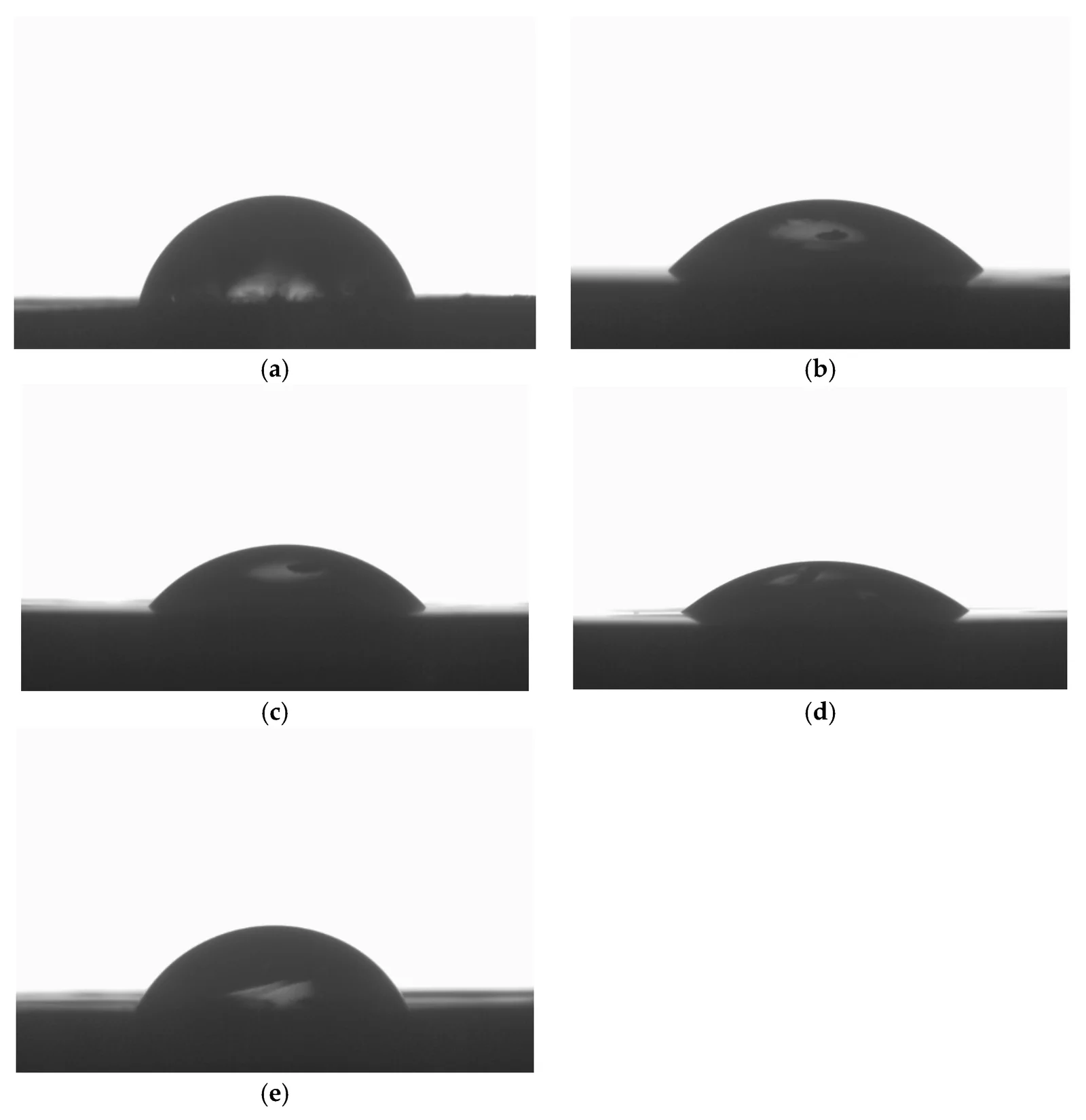
The images of deionized water droplets on the studied SLM surfaces obtained from the goniometry camera: (**a**) SLM0; (**b**) SLM1; (**c**) SLM2; (**d**) SLM3; and (**e**) SLM4.

The incorporation of QAHAMA-10 resulted in a significant (*p* < 0.0001) reduction in the mean *WCA* values (Figure 9). The highest *WCA* value was recorded for the reference material SLM0 (80.7°), whereas the lowest value was observed for SLM3 (48.6°). The images illustrating the *WCA* measurements of the tested SLMs are presented in Figure 10.

## 4. Discussion

The present study demonstrated that incorporation of QAHAMA-10 substantially improved the biological performance of the developed SLM materials by reducing their susceptibility to *Candida albicans* adhesion. This finding is particularly relevant because limiting the initial attachment of fungal cells to denture lining materials represents a critical step in preventing biofilm development and reducing the risk of denture stomatitis [28,29]. The antifungal activity of the investigated materials should be interpreted in relation to both their composition and the duration of water storage. After 24 h, the QAHAMA-10-modified formulations exhibited lower median CFU values than the reference material (SLM0). However, the overall differences among the groups did not reach statistical significance (Kruskal–Wallis test, *p* = 0.0785). Therefore, these findings indicate a numerical trend toward reduced fungal viability rather than a statistically confirmed effect. Furthermore, the observed changes were not strictly concentration-dependent, as SLM4 exhibited a higher median CFU value than SLM3. After 28 days of water storage, statistically significant overall differences among the formulations were observed (*p* = 0.0131). At this time point, the median CFU values were zero for SLM2, SLM3, and SLM4. Notably, no detectable CFUs were recorded for SLM3 and SLM4 in any of the reported measurements. These findings indicate favorable antifungal performance of the QAHAMA-10-modified materials after prolonged water storage, particularly for formulations containing higher concentrations of the functional monomer. However, the significant overall test result does not identify which individual formulations differed significantly, and a strictly concentration-dependent relationship cannot be established from the present data. Overall, the results suggest that the incorporation of QAHAMA-10 may contribute to improved antifungal properties, particularly after prolonged water storage. Nevertheless, the observed effects should be interpreted cautiously, considering the absence of statistically significant overall differences after 24 h and the simultaneous changes in QAHAMA-10 and MMA contents across the modified formulations. Additional microbiological analyses were performed to further elucidate the mechanism underlying the observed antifungal effect. The agar diffusion assay revealed that, for the investigated materials, no inhibition zones were observed, suggesting that the release of soluble active compounds into the surrounding medium, if present, was insufficient to produce a detectable antimicrobial effect under the tested conditions. However, this observation does not entirely exclude the possibility of limited release of active compounds and their localized effects within the microenvironment near the material surface. These findings may also be consistent with a possible contact-dependent mechanism, characteristic of dental polymers modified with polymerizable quaternary ammonium monomers [30,31]. Therefore, the absence of an inhibition zone should not be interpreted as a lack of antifungal efficacy, particularly in view of the observed reduction in fungal adhesion to the functionalized material surface. However, further release assays and complementary mechanistic studies are needed to clarify the potential contributions of localized release and contact-dependent effects.

Comparable observations have been reported for other dental polymers modified with polymerizable quaternary ammonium monomers, although these studies involved different classes of dental biomaterials rather than long-term soft denture lining materials. Mirizadeh et al. demonstrated that a polymethyl methacrylate denture base resin containing a quaternary ammonium monomer exhibited antimicrobial activity in direct contact assays despite the lack of inhibition zones in the agar diffusion test [31]. Similarly, Huang et al. reported comparable findings for an experimental Bis-GMA-free resin containing the polymerizable quaternary ammonium monomer IMQ-16, where pronounced antimicrobial activity was observed in contact-based assays despite the absence of inhibition zones [32]. Although these studies investigated denture base and restorative materials rather than soft denture liners, they support the interpretation that antimicrobial effects of polymers modified with quaternary ammonium monomers may be primarily contact-dependent and may not require the continuous release of active compounds. The antimicrobial mechanism of QAHAMA-10 is associated with the characteristic activity of quaternary ammonium compounds. Due to their permanent positive charge, these groups interact electrostatically with negatively charged components of microbial cell envelopes, leading to disruption of membrane integrity, increased permeability, impaired ion transport, and ultimately cell damage and lysis [33]. Because the quaternary ammonium groups are intended to be incorporated into the polymer network, this effect may persist during direct contact between microorganisms and the material surface without the need for significant leaching of active substances.

Notably, the pronounced reduction in *C. albicans* adhesion observed for SLM1–SLM4 occurred despite the increased surface hydrophilicity of the modified materials. Although hydrophilic surfaces may generally promote water retention, salivary protein adsorption, and initial microbial attachment, the present findings indicate that the contact-active mechanism of QAHAMA-10 may play a dominant role in controlling fungal adhesion [29,34]. Thus, in QAHAMA-10-modified soft liners, the antifungal effect appears to depend on factors beyond wettability alone, including the chemical functionality introduced by QAHAMA-10. The sustained reduction in fungal adhesion after 28 days further indicates that the antimicrobial functionality of QAHAMA-10 remained effective despite prolonged water storage and increased water sorption. This observation is clinically relevant because soft denture lining materials are continuously exposed to the oral environment during service, including saliva, temperature variations, and repeated microbial challenges [35]. Since *C. albicans* is considered the primary etiological factor associated with denture stomatitis, reducing its initial adhesion may limit subsequent biofilm maturation and decrease the risk of recurrent infection [36]. Therefore, incorporation of QAHAMA-10 may contribute to improved hygiene of denture liners by reducing fungal adhesion and potentially limiting biofilm accumulation during the intended temporary application.

The cytocompatibility assessment demonstrated that all tested materials maintained high cell viability throughout the incubation period, indicating good in vitro biological compatibility despite the incorporation of QAHAMA-10. According to ISO 10993-5 [37], materials are considered non-cytotoxic when cell viability exceeds 70%; therefore, all SLM formulations fulfilled this criterion. Importantly, the introduction of QAHAMA-10 did not result in biologically relevant cytotoxic effects, although a slight reduction in cell viability was observed for materials containing the highest monomer concentration. These results indicate that the incorporation of the quaternary ammonium monomer preserved the biological compatibility of the acrylic soft liner under the tested conditions. These findings are consistent with previous reports on quaternary ammonium methacrylate (QAM)-modified dental polymers. Ammar et al. reported that increasing concentrations of DMADDM enhanced antimicrobial activity but were also associated with a concentration-dependent reduction in cell viability, suggesting that excessive amounts of cationic monomers may negatively affect mammalian cells [38]. Similar observations have been described for other QAM-containing dental materials, where the strong positive charge responsible for antimicrobial activity may also influence interactions with eukaryotic cell membranes at elevated concentrations.

In the present study, the gradual incorporation of QAHAMA-10 enhanced the antifungal functionality while maintaining cell viability above the cytotoxicity threshold under the tested conditions. Despite the increased content of the cationic monomer, all modified materials maintained acceptable cell viability, suggesting that the concentrations applied were sufficient to introduce antifungal properties without compromising biological compatibility [9]. Therefore, QAHAMA-10 represents a potential modifier for acrylic soft liners, although its overall suitability remains to be established. In the present study, a clear effect of the modification of SLM composition on the physical, mechanical, and surface properties of the developed materials was demonstrated. The incorporation of QAHAMA-10, containing a quaternary ammonium group, resulted in a pronounced reduction in Shore A0 hardness compared with the unmodified material (SLM0), both before and after water conditioning. The reference material, which lacked this component, exhibited a more rigid polymer structure, whereas the QAHAMA-10-modified formulations showed a softer and more flexible character, which may be advantageous for temporary soft denture lining applications. The higher hardness of SLM0 may contribute to improved resistance to deformation and greater structural stability during use; however, excessive rigidity may negatively affect patient comfort, particularly in individuals with mucosal sensitivity, ridge atrophy, or compromised denture-bearing tissues [39]. In contrast, the lower hardness observed for the modified materials may facilitate stress distribution and improve cushioning of masticatory forces [40]. Importantly, the observed reduction in hardness was primarily associated with the incorporation of QAHAMA-10 rather than with a simple concentration-dependent relationship, as the modified materials exhibited similar mechanical profiles despite differences in monomer content.

The slightly higher initial hardness observed for SLM4 may be associated with additional ionic interactions within the polymer network at the highest monomer concentration [9]. Nevertheless, the hardness of SLM4 decreased after 28 days of water conditioning, indicating that prolonged exposure to the aqueous environment influenced the mechanical response of this formulation. This observation suggests that, despite the potential contribution of ionic interactions to the initial hardness increase, the long-term behavior of the material is also affected by environmental conditioning. Comparable findings were reported by Alsaiari et al. [41], who investigated silicone soft liners modified with ionic liquids, including butylpyridinium chloride (BPCL) and octylpyridinium chloride (OPCL). The hardness values reported in that study were comparable to those obtained for the reference material in the present work, whereas the QAHAMA-10-modified SLMs demonstrated a softer mechanical profile. This difference may be related to the distinct chemical composition of the investigated materials, as silicone-based liners exhibit different viscoelastic properties compared with the acrylic soft liners evaluated in the present study [41]. The tensile strength of the developed SLM formulations ranged from 0.79 to 0.99 MPa, falling within the range of approximately 0.12–1.35 MPa reported for commercially available temporary soft lining materials [42,43]. Although no widely accepted reference value for tensile strength has been established for these materials, the absence of adverse changes in this property is particularly important in the context of the present study [35].

The combination of reduced hardness and preserved tensile strength may be particularly advantageous for soft denture lining materials, as it may allow improved cushioning of functional loads while maintaining adequate mechanical stability during use [44,45]. Therefore, QAHAMA-10 modification appears to alter the mechanical profile of the material toward greater flexibility without compromising its essential strength characteristics. From a clinical perspective, the reduction in hardness induced by QAHAMA-10 may be beneficial, as softer materials can provide improved adaptation to resilient oral tissues, better cushioning of functional loads, and increased patient comfort [46]. However, maintaining an appropriate relationship between softness and mechanical stability remains important for the functional performance of temporary soft denture lining materials during their intended period of use [47]. The increased surface hydrophilicity of the QAHAMA-10-modified samples was reflected in reduced WCA values, which is consistent with previous reports describing the influence of cationic methacrylate monomers on surface wettability [48]. In the work by Ammar et al. [38], modification of a soft denture liner with the dimethylaminododecyl methacrylate (DMADDM) monomer likewise resulted in a pronounced decrease in contact angle with increasing monomer concentration, which the authors attributed to the presence of a hydrophilic, positively charged ammonium group incorporated into the polymer structure. Similarly, in the present study, the incorporation of QAHAMA-10 significantly altered the surface characteristics of the materials, resulting in increased hydrophilicity compared with the unmodified SLM0 [49]. This effect may be associated with the presence of quaternary ammonium groups and the increased capacity of the modified polymer surfaces to interact with water molecules through polar interactions and hydrogen bonding [50]. From a clinical perspective, increased surface wettability may be advantageous, as it can facilitate adaptation of the denture liner to the mucosa and support the formation of a stable salivary film, potentially improving retention and patient comfort [51]. Although highly hydrophilic surfaces may theoretically promote initial microbial attachment due to enhanced interactions with aqueous biological environments, this effect was not observed in the present study. The reduced *C. albicans* adhesion observed for QAHAMA-10-modified materials indicates that the contact-active antimicrobial properties of the incorporated quaternary ammonium groups may have influenced fungal adhesion despite the increased surface wettability. Therefore, in QAHAMA-10-modified soft liners, surface chemistry may play an important role in controlling fungal adhesion, although its contribution cannot be separated from that of wettability based on the present results. The literature emphasizes that enhanced wettability is often achieved artificially through surface treatments, such as plasma modification or the incorporation of hydrophilic agents; therefore, achieving this property as an intrinsic feature of the polymer structure may represent an additional advantage of QAHAMA-10 modification [52,53,54].

The *TBS* values obtained in the present study ranged from 0.25 to 0.40 MPa. The control formulation (SLM0) exhibited the highest mean *TBS* (0.40 MPa), whereas SLM4 showed the lowest value (0.25 MPa), representing a statistically significant reduction of approximately 37.5% compared with the control. These results are broadly comparable to those reported for acrylic-based temporary soft denture liners. Vuksic et al. reported *TBS* values of 0.19–0.25 MPa for an acrylic soft liner bonded to different denture base materials [55], while Li et al. reported values ranging from 0.09 to 0.50 MPa, depending on the material combination [56]. The lower *TBS* of SLM4 may be associated with changes in polymer composition and interfacial interactions resulting from the incorporation of QAHAMA-10 at the expense of other monomers. However, since the formulations differed in several compositional aspects, the reduction cannot be attributed exclusively to QAHAMA-10. Although the *TBS* of SLM4 was significantly lower than that of the control, its mean value remained within the range reported for soft denture relining materials in the literature [55,56].

The increased hydrophilicity of the QAHAMA-10-modified materials was accompanied by higher water sorption and solubility, which is an expected consequence of introducing a more polar monomer containing quaternary ammonium groups into the polymer structure [57,58]. SLM0 exhibited a more hydrophobic polymer structure with limited interaction with the aqueous environment, whereas the incorporation of QAHAMA-10 resulted in a greater affinity of the material matrix for water molecules [57]. This phenomenon can be attributed to the increased polarity of the monomer and the presence of hydrophilic ionic groups capable of interacting with water, leading to enhanced hydration of the polymer network. Similar effects have been reported for materials modified with quaternary ammonium compounds, where increased water uptake was associated with potential changes in mechanical stability over time [59]. In Bellusa’s study, the incorporation of quaternary-ammonium methacryloxy silicate into a hard lining material increased water uptake and could potentially compromise long-term mechanical stability, while simultaneously providing significant antimicrobial activity against *C. albicans* and oral streptococci [59]. Similarly, studies on dental resin composites containing quaternary ammonium monomers have reported changes in water sorption and solubility following the incorporation of these functional monomers. In the study by He et al. [60], the incorporation of bi-quaternary ammonium methacrylates into dental resin composites increased water sorption, although the obtained values remained within the applicable ISO requirements [61]. A similar balance between increased hydrophilicity and preserved functional performance was reported by Ammar et al., who demonstrated that DMADDM modification increased water affinity while simultaneously improving resistance to microbial colonization through a contact-killing mechanism [38]. These findings suggest that increased hydration of the polymer matrix may occur following the introduction of functional monomers without necessarily compromising antimicrobial activity or the mechanical properties evaluated under the investigated conditions. However, the preservation of the tested properties does not exclude potential effects on other material characteristics. The significance of increased water sorption and solubility should be interpreted in relation to the intended clinical application and their effects on other material properties and overall performance. Increased water sorption may affect dimensional stability and mechanical properties, whereas increased solubility may contribute to surface degradation and the release of residual or other extractable components, potentially affecting the biological performance and safety of the material. Unlike ISO 10139-2 [5], which specifies requirements for water sorption and solubility of long-term soft denture lining materials, ISO 10139-1 [4] does not establish corresponding acceptance criteria for temporary soft lining materials. Nevertheless, their temporary use does not eliminate the need to consider the potential consequences of increased water sorption and solubility. In the present study, despite increased water uptake in QAHAMA-10-modified materials, favorably low hardness values were maintained, indicating that the materials retained a relatively soft consistency. This effect appeared particularly beneficial for the formulations containing higher monomer concentrations (SLM3 and SLM4), which did not exhibit the progressive increase in hardness observed for the reference material during water storage. Moreover, SLM4 showed a slight decrease in hardness over time. Such behavior may be advantageous for temporary soft denture lining applications, as maintaining or further reducing rigidity during water conditioning may help preserve the cushioning function of the material [35]. In soft lining materials, increased water sorption and solubility may become clinically unfavorable when associated with the leaching of plasticizing components, resulting in progressive loss of elasticity, increased stiffness, and deterioration of cushioning properties during service [62]. In the present study, the observed hardness profile did not show this pattern of progressive hardening. However, hardness measurements alone cannot establish whether elasticity and other mechanical properties were preserved or exclude the release of residual monomers or other extractable components [63]. Therefore, the effects of QAHAMA-10 modification should be considered in terms of the balance between increased hydration of the polymer matrix and the potential functional benefits associated with improved surface properties and the maintenance of low hardness. Despite the promising results, several limitations of the present study should be acknowledged. First, the biological evaluation was performed using the L929 cell line; therefore, the obtained cytocompatibility results cannot be directly extrapolated to human oral tissues. Further studies involving clinically relevant human oral cell types are needed to confirm the biological safety of the developed materials. Second, the experimental design does not allow the individual contribution of QAHAMA-10 to be completely separated from the effects of other formulation changes. In the modified SLMs, QAHAMA-10 replaced BuMA, while the MMA content was progressively reduced and ethanol was introduced to dissolve QAHAMA-10. Therefore, the observed changes in material properties cannot be attributed exclusively to QAHAMA-10. Composition-matched control formulations would be required to independently determine the contribution of each formulation component and should be considered in future studies. Third, the antifungal activity was evaluated against *C. albicans*, which is highly relevant to denture-associated fungal infections, but the effectiveness of QAHAMA-10-modified SLMs against other clinically relevant microorganisms and mixed-species oral biofilms remains to be established [64]. Although the absence of inhibition zones is consistent with a possible contact-dependent antimicrobial mechanism, additional mechanistic studies would be required to directly confirm the interactions between the quaternary ammonium groups and microbial cell membranes [65,66]. Furthermore, the present study evaluated the stability and mechanical behavior of the materials after water conditioning for up to 28 days. Although this period exceeds the typical duration of use expected for short-term soft lining materials, water conditioning does not fully reproduce the complex conditions of the oral environment, including exposure to saliva, temperature fluctuations, mechanical loading, enzymatic activity, and repeated microbial challenges. Therefore, further studies under simulated oral conditions, including evaluation of adhesion to denture base materials and resistance to mechanical loading, would provide additional information regarding the clinical performance of the developed short-term soft lining materials [44]. Moreover, the increased water sorption and solubility nevertheless remain important limitations that warrant further investigation, particularly regarding component release, dimensional stability, and material durability during aging. Further formulation optimization should aim to reduce water sorption and solubility while preserving the desired softness and antifungal performance. Finally, in vivo and clinical studies are required to determine whether the antifungal activity, cytocompatibility, hydrophilicity, and mechanical properties observed in vitro can be maintained under actual conditions of use [67].

## 5. Conclusions

The incorporation of QAHAMA-10 improved the antifungal performance of the developed A-SLMs while maintaining acceptable cytocompatibility under the tested conditions. SLM3 exhibited the most favorable overall balance of these properties, achieving complete inhibition of recoverable *C. albicans* colonies under the applied test conditions while maintaining high cell viability, tensile strength comparable to values reported for other soft denture lining materials, and a consistently lower hardness compared with the reference material throughout the evaluation period. Compared with SLM4, SLM3 provided a more favorable overall balance of antifungal efficacy, cytocompatibility, mechanical performance, and surface properties, despite containing a lower concentration of QAHAMA-10 (15 wt.% vs. 18 wt.%). Both formulations completely inhibited recoverable *C. albicans* after 28 days of water conditioning; however, SLM3 also achieved complete inhibition after 24 h, whereas SLM4 showed a 72% reduction in *C. albicans* cell adhesion at this time point. Furthermore, SLM3 exhibited higher tensile strength, lower water solubility, and a lower water contact angle than SLM4, while both formulations met the cytocompatibility criterion under the tested conditions. These findings suggest that increasing the QAHAMA-10 content to 18 wt.% did not provide a sufficient additional antifungal benefit to outweigh the more favorable physicochemical profile of SLM3. Although QAHAMA-10 incorporation resulted in increased water sorption and solubility, these changes represent important limitations of the developed formulations, and their clinical significance cannot be determined from the present in vitro data. The incorporation of QAHAMA-10 also altered selected mechanical properties, indicating a trade-off between improved antifungal performance and the physicochemical and mechanical characteristics of the materials. Therefore, the initial hypothesis was only partially supported. Moreover, the increased surface wettability observed after QAHAMA-10 modification may influence interactions with the aqueous oral environment; however, its potential effects on clinical adaptation and patient comfort remain to be investigated. Importantly, this increased hydrophilicity did not compromise antifungal performance, as demonstrated by the microbiological evaluation. Overall, the results suggest that QAHAMA-10 may be a promising functional monomer for modifying acrylic soft denture lining materials intended for temporary use. However, these preliminary in vitro findings require further validation to establish the material’s biological safety, potential to limit biofilm development, and clinical relevance.

## Figures and Tables

**Table 1:** Chemicals, reagents, and monomers used in the study and relevant information on QAHAMA-10 synthesis.

Compound	Abbreviation	Function/Application	Relevant Details
Methyl methacrylate	MMA	Methacrylate monomer; synthesis of HAMA and formulation of liquid components	Acros Organics (Geel, Belgium)
N-Methyldiethanolamine	MDEA	Precursor for HAMA synthesis	
1-Bromodecane	BD	Alkylating agent for QAHAMA-10 synthesis	
Potassium carbonate	K_2_CO_3_	Catalyst in the transesterification step	POCH S.A. (Gliwice, Poland)
Toluene	TOL	Reaction medium for HAMA synthesis	
Chloroform	TCM	Extraction and purification solvent	
Phenothiazine	PTZ	Polymerization inhibitor	Sigma-Aldrich (St. Louis, MO, USA)
Ethylene glycol dimethacrylate	EGDMA	Crosslinking monomer	
N,N-Dimethyl-p-toluidine	DMPT	Polymerization accelerator	
Ethyl methacrylate	EMA	Methacrylate monomer in the liquid components	Acros Organics (Geel, Belgium)
Butyl methacrylate	BMA		
2-Phenethyl benzoate	PhB	Plasticizer	TCI (Tokyo, Japan)
Ethanol	EtOH	Solvent used to dissolve QAHAMA-10 and facilitate its incorporation into the experimental liquid components	Chempur (Piekary Śląskie, Poland)
Villacryl Soft powder	PC	Commercial powder component of the soft lining material, containing a methacrylate polymer and benzoyl peroxide (BPO) initiator	Everall7, (Warsaw, Poland)

**Table 2:** Chemical compositions of LCs.

LC	MMA [wt.%]	BuMA [wt.%]	QAHAMA-10 [wt.%]	ETOH [wt.%]	EGDMA [wt.%]	PhB [wt.%]	DMPT [wt.%] *
LC-0 (control)	7	11	0	0	2	80	1
LC-I	11	0	7	6	2	74	1
LC-II	7	0	11	6	2	74	1
LC-III	3	0	15	6	2	74	1
LC-IV	0	0	18	6	2	74	1

* DMPT content was calculated based on the total mass of the methacrylates used in each formulation: MMA + BuMA + EGDMA for LC-0 and MMA + QAHAMA-10 + EGDMA for LC-I–LC-IV.

**Table 3:** Antifungal efficacy of *Candida albicans* ATCC 2091 of the SLMs and Kruskal–Wallis test results (α = 0.05).

Time of Storage in Water	Parameters of Descriptive Statistics	Medians and Interquartile Ranges × 10^4^, CFU/mL				
		SLM0	SLM1	SLM2	SLM3	SLM4
24 h (*p* = 0.0785)	Med	12.7	2.8	1.9	0	3
	Min	1.9	2.4	0.1	0	2.6
	Max	24.8	24.7	3.6	0	5.5
	IQR	11.45	11.15	1.75	0	1.45
28 d (*p* = 0.0131)	Med	22.5	5	0	0	0
	Min	5.2	4.6	0	0	0
	Max	33	7	2.8	0	0
	IQR	13.9	1.2	1.4	0	0

**Table 4:** The results of one-way ANOVA and Tukey’s HSD post hoc tests for cell viability *.

Material Type	Incubation Time		
	24 h (*p* = 0.0321)	48 h (*p* = 0.0103)	72 h (*p* = 0.0147)
SLM0 (*p* = 0.0009)	-; b, c	E; a	B, C, D, E; a
SLM1 (*p* = 0.0706)	-; -	-; -	A; -
SLM2 (*p* = 0.0023)	-; b	-; a, c	A; -
SLM3 (*p* = 0.2756)	-; -	-; -	A; -
SLM4 (*p* = 0.0392)	-; c	A; -	A; a

* Uppercase letters (A–E) indicate comparisons between material groups within the same incubation period (column), whereas lowercase letters (a–c) indicate comparisons between incubation periods within the same material (row). Letters indicate statistically different values (α = 0.05) and refer to subsequent bars; a is identical to the first bar, b is identical to the second bar, etc. (Tukey’s HSD, α = 0.05).

**Table 5:** The results of one-way ANOVA and Tukey’s HSD post hoc tests for Shore A0 hardness *.

Material Type	Storage Time		
	24 h (*p* < 0.0001)	7 d (*p* < 0.0001)	28 d (*p* < 0.0001)
SLM0 (*p* < 0.0001)	B, C, D, E; b, c	B, C, D, E; a, c	B, C, D, E; a, b
SLM1 (*p* < 0.0001)	A, D, E; c	A, C, D, E; c	A; a, b
SLM2 (*p* < 0.0001)	A, D, E; b, c	A, B, E; a, c	A, D, E; a, b
SLM3 (*p* = 0.1518)	A, B, C, E; -	A, B, E; -	A, C; -
SLM4 (*p* < 0.0001)	A, B, C, D; b, c	A, B, C, D; a	A, C; a

* Uppercase letters (A–E) indicate comparisons between material groups within the same incubation period (column), whereas lowercase letters (a–c) indicate comparisons between incubation periods within the same material (row). Letters indicate statistically different values (α = 0.05) and refer to subsequent bars; a is identical to the first bar, b is identical to the second bar, etc. (Tukey’s HSD, α = 0.05).

## Data Availability

The original contributions presented in this study are included in the article/Supplementary Material. Further inquiries can be directed to the corresponding authors.

## Supplementary Materials

The following supporting information can be downloaded at: https://www.mdpi.com/article/10.3390/ma19204421/s1. Table S1: Signals characteristic of the QHAMA-C10 monomer in the ^1^H NMR spectrum. Table S2: Signals characteristic of the QHAMA-C10 monomer in the ^13^C NMR spectrum.
