# Supplementary material for: Development and Characterization of a Phthalate-Free Antifungal Soft Denture Lining Material Incorporating a Quaternary Ammonium Monomer
Source: Materials (Basel). 2026 Oct 21;19(20):4421. doi: 10.3390/ma19204421 (PMC13707371; doi:10.3390/ma19204421)
Supplement: Supplementary file 1 [file materials-19-04421-s001.zip › materials-4587865-supplementary.pdf]

Table S1. Signals characteristic of the QHAMA-C10 monomer in the ^{1}H NMR spectrum.

Signal Symbol	Structure	Multiplicity	Number of protons	Chemical shift [ppm]
a	$C\underline{\mathbf{H_3}}$-C=	s	3	1.92
b	=$C\underline{\mathbf{H_2}}$	2m	2	5.65 and 6.10
c	-O-$C\underline{\mathbf{H_2}}$-$CH_2$-$N^+$	m	2	4.65
d	-O-CH_2-$C\underline{\mathbf{H_2}}$-$N^+$	m	2	4.06
e	$C\underline{\mathbf{H_3}}$-$N^+$	s	3	3.39
f	HO-$C\underline{\mathbf{H_2}}$-$CH_2$-$N^+$	m	2	3.81
g	HO-CH_2-$C\underline{\mathbf{H_2}}$-$N^+$	m	2	4.13
h	-O$\underline{\mathbf{H}}$	bs	1	5.08
i	N^+-$C\underline{\mathbf{H_2}}$-$CH_2$-$(CH_2)_7$-$CH_3$	m	2	3.59
j	N^+-CH_2- $C\underline{\mathbf{H_2}}$-$(CH_2)_7$-$CH_3$	m	2	1.75
k	N^+-CH_2- CH_2-$(C\underline{\mathbf{H_2}})_7$-$CH_3$	m	14	1.22-1.30
l	N^+-CH_2- CH_2-$(CH_2)_7$-$C\underline{\mathbf{H_3}}$	t	3	0.87

Table S2. Signals characteristic of the QHAMA-C10 monomer in the ^{13}C NMR spectrum.

Signal Symbol	Structure	Chemical shift [ppm]
a	=C-$\underline{\mathbf{C}}H_3$	19
b	>$\underline{\mathbf{C}}$=	136
c	$\underline{\mathbf{C}}H_2$=	127
d	$\underline{\mathbf{C}}$=O	166
e	CH_3-$(CH_2)_8$-$\underline{\mathbf{C}}H_2$-$N^+$<br>-$\underline{\mathbf{C}}H_2$-$N^+$<br>-$\underline{\mathbf{C}}H_2$-O-	56 - 64
f	$\underline{\mathbf{C}}H_3$-$N^+$	51
g	CH_3-$(\underline{\mathbf{C}}H_2)_8$-$CH_2$-$N^+$	23 - 32
h	$\underline{\mathbf{C}}$H3-$(CH_2)_8$-CH_2-N^+	14
